# MicroRNAs as Potential Biomarkers for Chemoresistance in Adenocarcinomas of the Esophagogastric Junction

**DOI:** 10.1155/2019/4903152

**Published:** 2019-07-29

**Authors:** Christina Just, Juliana Knief, Pamela Lazar-Karsten, Ekaterina Petrova, Richard Hummel, Christoph Röcken, Ulrich Wellner, Christoph Thorns

**Affiliations:** ^1^Department of Pathology, Section of Hematopathology and Endocrine Pathology, University Hospital of Schleswig-Holstein, Campus Luebeck, Ratzeburger Allee 160, 23538 Luebeck, Germany; ^2^Department of Pathology, Marienkrankenhaus Hamburg, Alfredstrasse 9, 22087 Hamburg, Germany; ^3^Department of Surgery, University Hospital of Schleswig-Holstein, Campus Luebeck, Ratzeburger Allee 160, 23538 Luebeck, Germany; ^4^Department of Pathology, University Hospital of Schleswig-Holstein, Campus Kiel, Arnold-Heller-Strasse 3, 24105 Kiel, Germany

## Abstract

Concerning adenocarcinomas of the esophagogastric junction, neoadjuvant chemotherapy is regularly implemented, but patients' response varies greatly, with some cases showing no therapeutic effect, being deemed as chemoresistant. Small, noncoding RNAs (miRNAs) have evolved as key players in biological processes, including malignant diseases, often promoting tumor growth and expansion. In addition, specific miRNAs have been implicated in the development of chemoresistance through evasion of apoptosis, cell cycle alterations, and drug target modification. We performed a retrospective study of 33 patients receiving neoadjuvant chemotherapy by measuring their miRNA expression profiles. Histologic tumor regression was evaluated using resection specimens, while miRNA profiles were prepared using preoperative biopsies without prior therapy. A preselected panel of 96 miRNAs, known to be of importance in various malignancies, was used to test for significant differences between responsive (chemosensitive) and nonresponsive (chemoresistant) cases. The cohort consisted of 12 nonresponsive and 21 responsive cases with the following 4 miRNAs differentially expressed between both the groups: hsa-let-7f-5p, hsa-miRNA-221-3p, hsa-miRNA-31-5p, and hsa-miRNA-191-5p. The former 3 showed upregulation in chemoresistant cases, while the latter showed upregulation in chemosensitive cases. In addition, significant correlation between high expression of hsa-miRNA-194-5p and prolonged survival could be demonstrated (*p* value <0.0001). In conclusion, we identified a panel of 3 miRNAs predicting chemoresistance and a single miRNA contributing to chemosensitivity. These miRNAs might function as prognostic biomarkers and enable clinicians to better predict the effect of one or more reliably select patients benefitting from (neoadjuvant) chemotherapy.

## 1. Introduction

Since the discovery of microRNAs (miRNAs), which are small, noncoding RNAs with a length of 19–22 nucleotides [1], multiple studies have focused on the importance of their function and participation in human diseases ranging from inflammatory disorders and autoimmune diseases to malignant tumors including melanoma, various epithelial cancers, and hematological malignancies [2]. miRNAs have increasingly been used not only as diagnostic but also as prognostic biomarkers, and several studies have suggested the existence of tissue-specific miRNA signatures which might be used to classify different cancer types [3–5].

Regarding human cancer in general, miRNAs have been found to act not only as oncogenes, promoting tumor growth and dissemination, but also as tumor suppressors, inhibiting tumor cell proliferation and migration and inducing apoptosis [6]. Sometimes, their function varies in a single organ, showing divergent behavior in specific histologic tumor types (for example, adenocarcinoma vs. squamous cell carcinoma of the esophagus) [7].

Concerning carcinomas of the upper gastrointestinal tract, especially gastric cancer and adenocarcinoma of the esophagogastric junction, a multitude of miRNAs have been identified as useful biomarkers: for example, upregulation of miRNA-17-5p, miRNA-20a, miRNA-106b, miRNA-150, and miRNA-93 has been reported to inhibit apoptosis and to promote cell cycle progression, whereas downregulation of miRNA-29 and miRNA-375 has been shown to increase cell growth and migration [4, 8]. Other commonly dysregulated miRNAs include miRNA-21 and miRNA-19a/b which both promote lymph node and distant metastases as well as invasion of blood vessels when overexpressed [4]. In addition, a seven-miRNA panel consisting of miRNA-10b, miRNA-21, miRNA-223, miRNA-338, let-7a, miRNA-30a-5p, and miRNA-126 has been found to reliably predict survival in gastric cancer patients and is related not only to overall but also relapse free survival [9]. Besides, various miRNAs have been found to be associated with poor survival in both gastric carcinomas and adenocarcinomas of the esophagogastric junction, including miRNA-16, miRNA-21, miRNA-29, miRNA-125b, miRNA-130a, miRNA-141, miRNA-203a, miRNA-222, miRNA-302c, and miRNA-451 [8, 10–15].

Complementing their diagnostic and prognostic significance, miRNAs have also been found to contribute to chemoresistance and/or chemosensitivity via regulation of apoptosis, DNA damage, and repair mechanisms, and epithelial-mesenchymal transition and modulation of drug targets, drug-metabolizing enzymes, and drug efflux transporters [16, 17]. Especially in gastric cancer, the following miRNAs (amongst others) have been found to substantially contribute to chemoresistance: let-7b, miRNA-106a, miRNA-142, miRNA-143, miRNA-21, miRNA-338, miRNA-340, miRNA-497, miRNA-503, and miRNA-582—their target genes including *PTEN* (phosphatase and tension homolog), *BCL*_*2*_ (B-cell lymphoma 2), and IGF1R (insulin-like growth factor 1 receptor) [18–25]. For esophageal (adeno-) carcinoma, miRNA-141, miRNA-148a, miRNA-200c, miRNA-221, miRNA-27a, and miRNA-296 are said to contribute to chemoresistance [26]. This miR-mediated chemoresistance is mainly directed against commonly used therapeutic agents such as cisplatin, 5-fluorouracil, and vincristine [27].

Following these observations and taking into account that data concerning chemoresistance in carcinomas of the esophagogastric junction are still scarce and many studies have focused on cell lines rather than tissue specimen, our study aimed to further contribute to the knowledge for this entity by comparing miRNA profiles in chemoresistant and chemosensitive cancer tissue.

## 2. Materials and Methods

### 2.1. Selection of Cases

Samples from formalin-fixed, paraffin-embedded (FFPE) tissue containing adenocarcinomas of the esophagogastric junction were included in the present study. All patients received neoadjuvant chemotherapy. Only tumor-containing samples of preoperative biopsies (without prior therapy) were used for miRNA analysis, while whole-resection specimens (postchemotherapeutic) were taken to determine the degree of histological regression and thereby treatment response. All cases were collected as part of routine clinical care at the University Hospital of Schleswig-Holstein, Campus Luebeck, during 1997–2013. All analyses performed were in accordance with the Declaration of Helsinki and had been approved by the local Ethics Committee beforehand (reference number 14-242A).

### 2.2. Histologic Examination

Samples were carefully examined by two researchers (CJ and JK) with a light microscope (Axioskop, Zeiss, Jena, Germany), and histologic tumor types were determined using the current WHO standard [28]. Regression after chemotherapy was determined using haematoxylin- and eosin-(H&E-) stained slides and rated according to the system devised by Becker et al. [29]. Regression grades 1a and 1b were considered as having responded to therapy (responder group), while regression grade 3 was considered nonresponsive (nonresponder group). Cases with regression grade 2 were not included in the study as an assignment to either group could not reliably be undertaken (partial response).

### 2.3. RNA Isolation and miRNA Profiling

RNA for profiling of miRNA was isolated from FFPE tissue using the RecoverAll™ total nucleic acid isolation kit (Applied Biosystems, Carlsbad, California, USA). RNA concentrations were quantified using the NanoDrop Spectrophotometer (Nanodrop Technologies, Montchanin, New Castle, Delaware, USA). Afterwards, reverse transcription (RT) using amounts of 20 ng of total RNA by applying the miRCURY LNA™ Universal cDNA Synthesis Kit II (Exiqon, Vedbaek, Denmark), containing synthetic RNA Spike Ins, was performed. 5 *μ*l of the RT products was combined with the PCR master mix and nuclease-free water from the miRCURY LNA™ ExiLENT SYBR® Green master mix (Exiqon, Vedbaek, Denmark). After that, 10 *μ*l of the PCR Master mix-cDNA mix was added to each 384-well plate of the miRCURY LNA™ Universal RT miR Ready-to-Use PCR, Cancer focus panel, V4 (Exiqon, Vedbaek, Denmark). Finally, qPCR was performed by using the LightCycler® 480 instrument (Roche molecular systems Inc., Mannheim, Germany). All reactions were carried out according to the manufacturer's instructions. Initial data analysis was executed by using the LightCycler® 480 Software (Roche molecular systems Inc., Mannheim, Germany) to obtain raw Ct values. Ct values were used to determine the amount of miRNA in a sample (both parameters showing an inverse correlation).

### 2.4. Preprocessing of Data

In order to compensate for variations in quality of extracted RNA, extraction yield, and efficiency of reverse transcription, normalization of data was carried out using GenEx Software Version 6.1 (Trial Version; MultiD Analyses AB, Munich). The first normalization method used was the Normfinder algorithm which has been described in detail earlier [30]. In our analysis, a panel of 38 miRs from the data set and a single miR (hsa-miRNA-103a-3p) were selected for preprocessing as hsa-miRNA-103a-3p was stably expressed throughout the cohort and has been reported as being highly reliable for normalization of data [31]. Secondly, external controls (so-called Spike Ins—preformulated, commercially available RNAs with defined lengths and binding capacities), which were included in the beginning as potential references, were used to normalize the data [32]. The last normalization method used was the global mean algorithm which—in three steps—reduces nonspecific background noise, calculates the arithmetic mean value for all samples, and then subtracts this value from each individual value [33]. The global mean method is usually applied in cases where large numbers of miRNAs are tested and has been reported to be superior to other normalization methods in this particular setting [34].

### 2.5. Analysis of Subgroups

To test for significant differences in miRNA expression profiles between responder and nonresponder groups, the Mann–Whitney-*U* test for unpaired samples was applied using SPSS Statistics Version 22 (IBM, Ehringen, Germany). Because exploratory data analysis was performed, adjusting for multiple testing was not required. Afterwards, overlaps of differentially expressed miRNAs between the applied normalization procedures described above were compared.

### 2.6. Correlation with Clinical Characteristics

To estimate differences in clinical features (age, gender, differentiation grade, nodal status, depth of infiltration, perineural invasion, lymphovascular invasion, and presence of distant metastases) between both groups and between clinical features and miRNA expression levels, the *χ*^2^ test was applied and a *p* value <0.05 was considered statistically significant.

### 2.7. Correlation with Overall Survival

To assess the prognostic value of miRNA expression, the median for each analyzed miRNA was calculated. Cases were then dichotomized, either showing an expression level above or below the median as described previously [35]. Overall survival curves were visualized via Kaplan–Meier estimates using SPSS Statistics Version 22 (IBM, Ehringen, Germany). In addition, Cox regression analysis was used to test for independence, taking into consideration gender, depth of infiltration, differentiation grade, nodal status, and presence or absence of distant metastases.

Data were adjusted for multiple testing using the Bonferroni procedure; after that, a *p* value <0.00052 was considered statistically significant for this test.

## 3. Results

### 3.1. Histology

**Figure 1:**
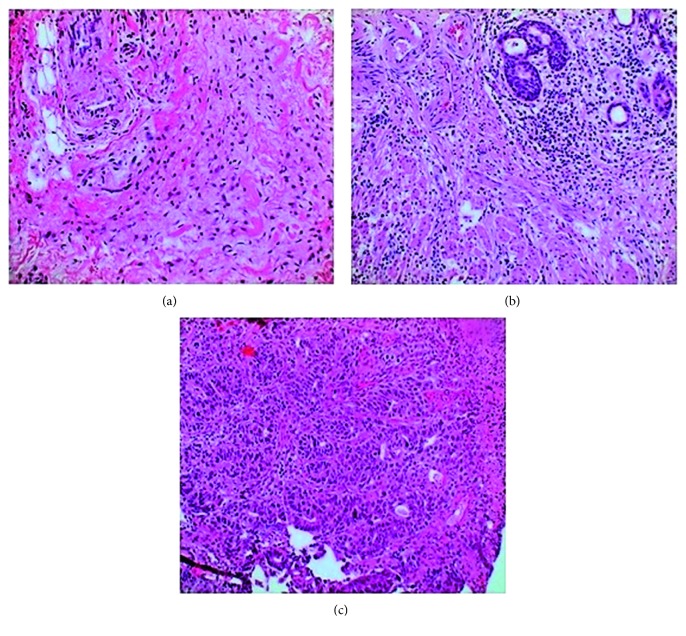
Pictures of histologic tumor regression: (a) regression grade 1a (no vital tumor); (b) regression grade 1b (<10% vital tumor cells); (c) regression grade 3 (>50% vital tumor cells). H&E staining, magnification 200x.

Overall, 24 cases were classified as tubular adenocarcinoma, 3 cases as poorly cohesive carcinoma, 2 cases as mucinous adenocarcinoma, and 4 cases as undifferentiated/unclassifiable according to the current WHO standard [36]. After thorough histologic examination of whole-resection specimens, the cohort consisted of 12 nonresponders (regression grade 3) and 21 responders (regression grades 1a and 1b). Tumor regression was determined as mentioned above; representative examples of regression grading are shown in Figure 1. Further characteristic features of the study cohort are summarized in Table 1.

### 3.2. Correlation with Clinical Characteristics

Correlation between both groups showed that undifferentiated carcinomas and poorly differentiated carcinomas (differentiation grade 3) were more common in the nonresponder group, while responders showed a higher proportion of tubular adenocarcinomas (*p* values 0.034 and 0.043, respectively). No differences could be detected concerning gender, depth of infiltration, lymphovascular/perineural invasion, nodal status, or presence of distant metastases (*p* values between 0.087 and 0.443, as shown in Table 1).

### 3.3. Therapy Regimen

Neoadjuvant chemotherapy was administered in all cases, and 3 patients received radiation therapy in addition (both belonging to the responder group with irradiation doses of 45 Gy, 59 Gy, and 66 Gy, respectively).

Concerning the nonresponder group, neoadjuvant chemotherapy consisted in most cases of 5-fluorouracil combined with leucovorin, oxaliplatin, and docetaxel (so-called FLOT regimen; 8/12 patients). The remaining patients received either 5-fluorouracil in combination with cisplatin (2 cases) or epirubicin combined with oxaliplatin (2 cases).

In the responder group, a combination of cisplatin and 5-fluorouracil was administered in most cases (12/21 patients). Only 4 patients received treatment using the FLOT regimen, and 2 were treated with 5-fluorouracil in combination with leucovorin and etoposide. The remaining three patients received chemotherapy without 5-fluorouracil containing oxaliplatin, etoposide, and irinotecan.

### 3.4. miRNA Analysis

Overall, the following 4 miRNAs were differentially expressed between responder and nonresponder groups: hsa-let-7f-5p, hsa-miRNA-191-5p, hsa-miRNA-221-3p, and hsa-miRNA-31-5p.

Concerning the different normalisation methods, only minor variations were detected: applying the Normfinder algorithm, hsa-let-7f-5p, hsa-miRNA-191-5p, and hsa-miRNA-31-5p were differentially expressed (*p* values 0.03, 0.005, and 0.047). For normalisation with Spike Ins, differences in expression of hsa-miRNA-221-3p and hsa-miRNA-31-5p could be observed (*p* values 0.022 and 0.02). For normalisation with global mean, hsa-let-7f-5p, hsa-miRNA-191-5p, hsa-miRNA-221-3p, and hsa-miRNA-31-5p were differentially expressed (*p* values 0.025, 0.014, 0.04, and 0.033, respectively). Finally, for normalisation using hsa-miRNA-103a-3p, hsa-let-7f-5p and hsa-miRNA-31-5p showed differences in expression (*p* values 0.038 and 0.036).

**Figure 2:**
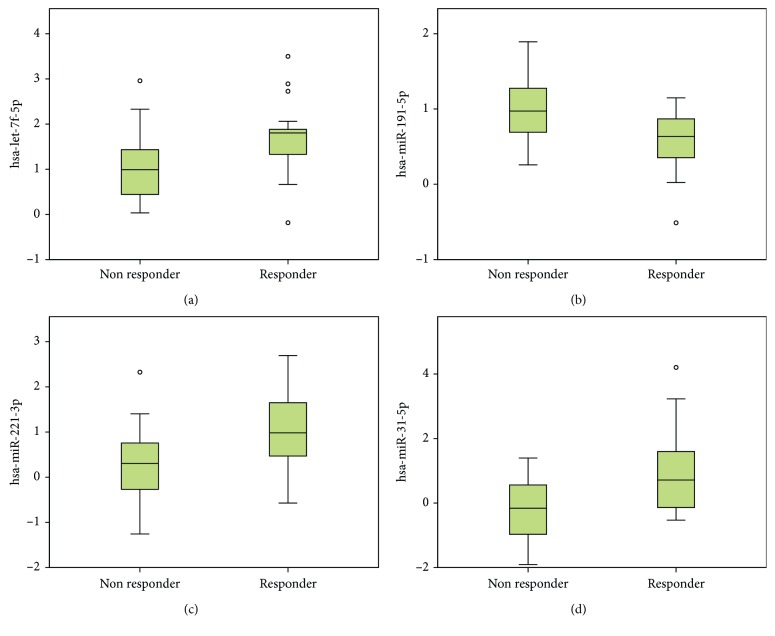
Boxplots and Ct values according to differentially expressed miRNAs in responder and nonresponder groups: (a) Ct values for hsa-let-7f-5p; (b) Ct values for hsa-miRNA-191-5p; (c) Ct values for hsa-miRNA-221-3p; (d) Ct values for hsa-miRNA-31-5p. Lower Ct values indicate higher miR expression, while higher Ct values indicate lower miR levels.

Throughout all normalization methods, there was higher expression of hsa-let-7f-5p, hsa-miRNA-221-3p, and hsa-miRNA-31-5p in the nonresponder group, while hsa-miRNA-191-5p showed higher expression in the responder group. The respective miRNA expression patterns (Ct values) are depicted in Figure 2.

### 3.5. miRNAs and Tumor Differentiation Grade

Correlation between miRNA expression profiles and tumor differentiation grade found that poorer differentiation (G3) was significantly associated with decreased levels of hsa-miRNA-200a-3p and elevated levels of hsa-miRNA-21-5p, hsa-miRNA-222-3p, hsa-miRNA-25-3p, and hsa-let-7d-5p (*p* values 0.03–0.031).

### 3.6. miRNAs and Patients' Prognosis

Complete survival data were available for 29 patients with a mean follow-up period of 44.52 months (range 1–100 months). During follow-up, 12 patients (41.38%) died. After adjusting for multiple testing, significant differences in survival according to high or low expression of miRNAs were detected only for hsa-miRNA-194-5p with a *p* value <0.0001. Survival times in patients with higher expression were nearly three times longer than in those with low expression (97.67 months vs. 32.69 months). Cox regression analysis, however, could not show independence for prediction of patients' survival after taking into consideration gender, depth of infiltration, differentiation grade, nodal status, and presence or absence of distant metastases (*p*=0.462; hazard ratio 1.469; 95% confidence interval 0.527–4.096). In addition, there was no correlation between expression levels of hsa-miRNA-194-5p and drug response (*p*=0.456).

**Figure 3:**
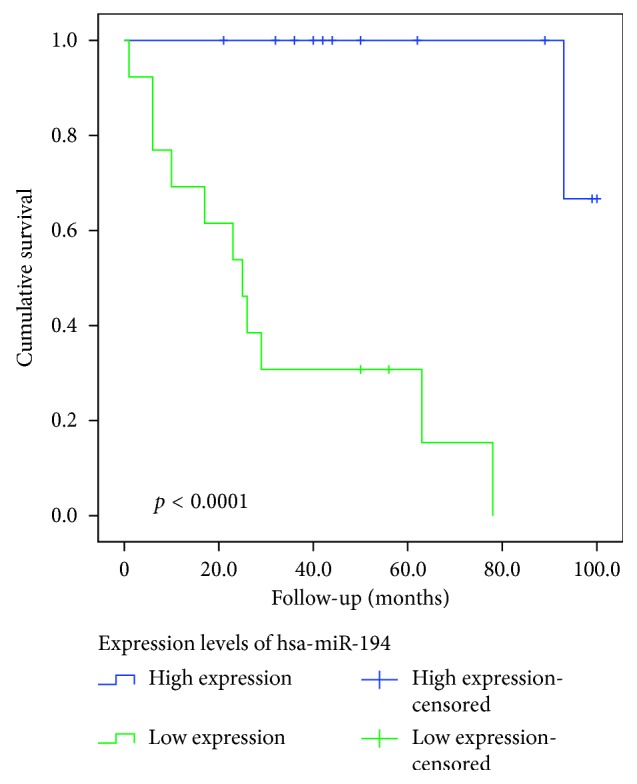
Kaplan–Meier curve showing survival differences according to high or low expression of hsa-miRNA-194-5p with a *p* value <0.0001. Survival times are given in months.

Appropriate survival curves and mean survival times including 95% confidence interval are shown in Figure 3 and Table 2.

## 4. Discussion

The mechanisms underlying chemotherapy and multidrug resistance in human cancer are polymorphic [37]. In this context, miRNAs have been found to regulate apoptosis, DNA repair, and epithelial-mesenchymal transition and to modulate drug targets, drug-metabolizing enzymes, or drug efflux transporters [16, 17]. In our study, analyzing adenocarcinomas of the esophagogastric junction, we identified a panel of four miRNAs which were differentially expressed between patients responding or not responding to neoadjuvant chemotherapy. We found higher expression of hsa-let-7f-5p, hsa-miRNA-221-3p, and hsa-miRNA-31-5p in the nonresponder group, while hsa-miRNA-191-5p showed higher expression in the responder group. The molecular mechanisms contributing to chemoresistance or chemosensitivity concerning these four miRNAs are—up to date—not fully understood. Nevertheless, the function and effects of these miRNAs as stated in the literature might give some clues as to what the underlying mechanisms might be: for hsa-let-7f-5p, a proangiogenic effect has been reported; thus, overexpression might contribute to tumor progression via tumor neoangiogenesis [38–40]. For hsa-miRNA-221-3p, cell cycle regulation has been reported as a key mechanism in tumor progression; in addition, in cervical cancer, increased expression levels are associated with epithelial-mesenchymal transition, migration, and invasion by targeting TWIST2 [41, 42]. For human glioblastomas, cervical and colon carcinoma cells downregulation of *PTEN* and activation of Akt and STAT3—mediated by increased levels of hsa-miRNA-221-3p—have been shown as key players in tumor cell survival and radio- and chemoresistance [43–46]. In addition, in hepatocellular carcinoma, upregulation of hsa-miRNA-221-3p decreases the expression of HDAC6, a tumor suppressor, and promotes tumorigenesis [47]. One study focusing solely on esophageal adenocarcinomas showed that the chemoresistance was conveyed through alteration of the Wnt/*β*-catenin pathway and DKK2, CDH1, CD44, MYC, and ABCG2 expression [26]. Concerning hsa-miRNA-31-5p, only few studies have addressed how increased expression contributes to chemoresistance: in malignant pleural mesothelioma and hepatocellular carcinoma, it promotes chemoresistance by targeting OCT1 and ABCB9 [48, 49]. In ovarian cancer, chemoresistance is increased by modulation of specific calcium-regulated potassium channels [50]. Conversely, an opposite effect has also been reported: overexpression of hsa-miRNA-31-5p decreases levels of stathmin 1, a microtubule-depolymerizing molecule that leads to reduced chemosensitivity in ovarian cancer [51]. In addition, in osteosarcoma, upregulation of hsa-miRNA-31-5p inhibits tumor cell migration and invasion by targeting PI3K3C2A [52]. Regarding hsa-miRNA-191-5p, the few studies conducted so far show that overexpression promotes chemoresistance by modulating p53 and TET1 in cholangiocarcinomas [53]. Furthermore, an association with various estrogen-dependent genes such as ANXA1, PIWIL2, CASP4, ESR1/ESR2, PLAC1, and SOCS2, has been shown in breast cancer—however, up to date, no such data concerning adenocarcinomas of the esophagogastric junction have been published [54].

The miRNA signature we discovered seems to differ from previously published data; other studies found that especially in esophageal carcinoma—in addition to miRNA-221—miRNA-141, miRNA-200c, miRNA-148a, miRNA-296, miRNA-23, miRNA-223, and miRNA-27a are substantially contributing to chemoresistance [26, 55]. Nevertheless, as some studies focus on plasma-circulating miRNAs or cell lines and not exclusively on expression in tumor tissue, results are comparable only to a limited degree [13]. Ours is—to our knowledge—the first study focusing on miRNA expression profiles in adenocarcinomas of the esophagogastric junction and their meaning for therapeutic response based on tissue specimens.

Hsa-let-7f-5p is located on the long arm of chromosome 9 (9q22.3) and shows involvement in immune cell differentiation, angiogenesis, and cellular growth arrest [56–58]. It is commonly affected in multiple human cancers, including melanoma, lung, and head/neck cancer, with downregulation in the majority of cases [59–61]. Nevertheless, upregulation is also encountered, for instance, in papillary, follicular, and anaplastic thyroid cancer as well as breast cancer and ovarian cancer [62–64]. It has been associated not only with chemosensitivity and treatment response in gastric cancer [65] but also with chemotherapy resistance in breast cancer [37]. In our study, increased expression of hsa-let-7f-5p showed a distinctive association with chemoresistance which seems to be in contrast to previously published data concerning carcinomas of the upper gastrointestinal tract [65]. Still, as data concerning the association of miR expression levels and chemotherapy response are still often controversial and sometimes different functions (either as a tumor suppressor or as an oncogene) have been reported for different histologic tumor types (adenocarcinoma vs. squamous cell carcinoma) in a single organ, our data might only mirror a specific effect for a defined subset of patients [7].

Hsa-miRNA-221-3p is well characterized, and its function and involvement in human cancer has been extensively described. It is commonly known as an onco-miRNA, promoting tumor proliferation, invasion, dissemination, and metastasis [66, 67]. Multiple analyses have focused on cell lines where overexpression is commonly associated with chemoresistance and knockdown restores chemo- and/or radio sensitivity and induces tumor cell apoptosis [26, 68, 69]. In addition, in tissue experiments, hsa-miRNA-221-3p has been shown to promote resistance to a variety of regularly used therapeutic agents including 5-fluorouracil, tyrosine kinase inhibitors, and antiandrogens [37, 70–73].

These findings are difficult to compare with our study population as both responders and nonresponders had been treated using a combination chemotherapy containing 5-fluorouracil in most cases (28/33 cases).

Downregulation of hsa-miRNA-31-5p has been described in a variety of human cancers, for instance, triple-negative breast carcinoma [67] and indicates shortened overall survival in gastric cancer [74] as well as the presence of locally advanced tumor stages, implicating the function as a miRNA with tumor suppressor properties.

Data concerning its association with therapy response are more controversial: while some studies report that overexpression promotes chemoresistance in gastric and ovarian cancer [50, 75] and breast carcinomas [76], others could demonstrate that upregulation in gallbladder carcinomas leads to increased chemosensitivity [77]. In addition, hsa-miRNA-31-5p expression levels seem to influence the therapy response not only in general but also according to different chemotherapeutic agents: in cell line experiments, it has been demonstrated that downregulation can promote resistance to platinum-based therapies and paclitaxel [78], while upregulation correlates with resistance against 5-fluorouracil [79]. Our study adds to the current understanding as we could demonstrate increased chemotherapy resistance in cases with higher hsa-miRNA-31-5p expression levels. It seems as if the therapeutic response might not only depend on the tumor subtype analyzed but also on the therapy applied and that hsa-miRNA-31-5p might exhibit both oncogenic and tumor suppressive functions according to a specific context.

Throughout the literature, hsa-miRNA-191-5p is described as having oncogenic properties, leading to increased tumor cell proliferation, invasion, and inhibition of apoptosis [80]. This holds true for various epithelial cancer types such as breast, pancreatic, and hepatocellular carcinomas [81] or cholangiocarcinoma. Here, overexpression is additionally associated with decreased overall survival [53]. Concerning its association with chemotherapy outcome, single studies have found hsa-miRNA-191-5p to promote chemoresistance [54], while others could show no influence on chemosensitivity or chemoresistance at all [82]. Overall, literature addressing this particular issue is still very sparse. In contrast to the data described above, in our study, high levels of hsa-miRNA-191-5p showed a clear-cut association with chemosensitive cases responding to neoadjuvant chemotherapy.

It remains to be seen whether these results can be reproduced in larger studies or if a similar effect can be shown in other cancer entities. It may be conceivable that our findings reflect—as it is possibly the case with both hsa-let-7f-5p and hsa-miRNA-31-5p—an effect which is only discernible in a defined subset of tumors or specific tumor entities.

Due to the small case number in our study and the often limited availability of both pretherapeutic biopsies and resection specimen in a single institution, we additionally consulted a web database (GEO database) to supplement our data. Here, we could find only one additional study analyzing chemotherapy response in a very small cohort (8 cases) of upper gastrointestinal carcinomas, namely, stomach cancer, proposing a miRNA signature used for predicting chemotherapy outcome [65]. This signature, however, differed from our findings, showing an association of chemoresistance and high expression of let-7g, miR-342, miR-16, miR-181, miR-1, and miR-34. Whether this might be due to small case numbers in both studies or whether this reflects differences between two cancer entities with a fundamentally different pathophysiology remains to be seen. Another study analyzed esophageal adenocarcinomas (14 cases) and reported that miRNA-221 contributes to chemoresistance via the Wnt/*β*-catenin pathway, supporting our findings [26].

In addition, we correlated the miRNA expression with patients' overall survival and could demonstrate that overall survival was significantly correlated with expression levels of hsa-miRNA-194-5p (*p* value <0.0001) although independence could not be demonstrated applying Cox regression analysis (*p*=0.462; hazard ratio 1.469; 95% confidence interval 0.527–4.096). In accordance with our results, where higher levels of hsa-miRNA-194-5p corresponded to prolonged survival (97.67 months vs. 32.69 months), overexpression has been reported to inhibit cell proliferation and to act as a tumor suppressor in laryngeal SCC, prostate cancer, melanoma, bladder cancer, NSCLC, and clear cell renal carcinoma [83–87]. In addition, overexpression has been found to inhibit growth and proliferation in gastric cancer cell lines and esophageal squamous cell carcinoma [88, 89]. Other miRNAs which are commonly linked to prolonged or shortened overall survival in gastric or esophageal carcinomas—for instance, miRNA-16, miRNA-21, miRNA-29, miRNA-125b, miRNA-130a, miRNA-141, miRNA-203a, miRNA-222, miRNA-302c, and miRNA-451—could not be reproduced in our study [4, 8, 9, 11, 14]. This might well be due to a smaller number of patients included in our study which limits the statistical reliability as well as the fact that, after adjusting for multiple testing, only a *p* value <0.00052 was considered statistically significant which excluded a few otherwise statistically significant miRNAs.

## 5. Conclusion

Our results imply that miRNAs might play an important role in the evolution of chemoresistance and/or chemosensitivity in adenocarcinomas of the esophagogastric junction. Nevertheless, as—due to the restricted availability of both pre- and posttherapeutic tissue samples in a single institution—the number of patients in our study was limited, statistical results should be interpreted with caution. In addition, only tumor tissue was compared without establishing baseline levels of miRNA expression in nonneoplastic mucosa.

Regardless of the abovementioned limitations, our results contribute to other studies postulating that miRNAs might be a pretherapeutic means to predict therapy response in the future and better stratify patients who benefit from neoadjuvant therapy or who might not benefit at all and therefore could be spared of adverse effects of noneffective treatment strategies.

It remains to be seen if the miRNA signature we established for adenocarcinomas of the esophagogastric junction can be reproduced in future studies or for different tumor entities.

## Figures and Tables

**Table 1:** Characteristics of adenocarcinoma of the esophagogastric junction according to the responder or nonresponder status.

Characteristics	Responders	Nonresponders	*p* value
Total *n*	21	12	
Gender			
Male	18	8	0.198
Female	3	4	
WHO classification			
Tubular	17	7	**0.034**
Poorly cohesive	2	1	
Mucinous	2	0	
Undifferentiated	0	4	
Others			
Differentiation grade			
Well (G1)	0	0	**0.043**
Moderately (G2)	19	7	
Poor (G3)	2	5	
pT (low)			
pT0	2	0	0.136
pT1a	2	0	
pT1b	5	0	
pT2	3	1	
pT (high)			
pT3	8	10	
pT4a	1	1	
pT4b	0	0	
pN			
pN0	12	6	0.203
pN1	3	4	
pN2	6	1	
pN3	0	1	
*LVSI*			
Present	6	5	0.443
Absent	15	7	
Perineural invasion			
Present	1	3	0.087
Absent	20	9	
Distant metastases			
Present	4	1	0.409
Absent	17	11	

LVSI: lymphovascular space invasion; bold lettering in *p* values indicates a statistically significant difference.

**Table 2:** Average survival times according to high or low expression of hsa-miRNA-194-5p.

	Average survival (months)	SD	95% CI	*p* value
hsa-miRNA-194-5p				
High	97.67	1.91	93.93–101.4	**<0.0001**
Low	32.69	7.89	17.22–48.16	

## Data Availability

The data used to support the findings of this study are available from the corresponding author upon request.

## Abbreviations

Ct: Cycle threshold
hsa: *Homo sapiens*
miRNA: MicroRNA.
